# Molecular sampling of prostate cancer: a dilemma for predicting disease progression

**DOI:** 10.1186/1755-8794-3-8

**Published:** 2010-03-16

**Authors:** Andrea Sboner, Francesca Demichelis, Stefano Calza, Yudi Pawitan, Sunita R Setlur, Yujin Hoshida, Sven Perner, Hans-Olov Adami, Katja Fall, Lorelei A Mucci, Philip W Kantoff, Meir Stampfer, Swen-Olof Andersson, Eberhard Varenhorst, Jan-Erik Johansson, Mark B Gerstein, Todd R Golub, Mark A Rubin, Ove Andrén

**Affiliations:** 1 Department of Molecular Biophysics and Biochemistry, Yale University, New Haven, Connecticut, 06520, USA; 2 Department of Pathology and Laboratory Medicine, Weill Cornell Medical Center, New York, New York, USA; 3 Institute for Computational Biomedicine, Weill Cornell Medical Center, New York, New York, USA; 4 Department of Medical Epidemiology and Biostatistics, Karolinska Institutet, Stockholm, Sweden; 5 Department of Biomedical Sciences and Biotechnologies, University of Brescia, Brescia, Italy; 6 Department of Pathology, Brigham and Women's Hospital, Boston, Massachusetts, 02115, USA; 7 The Broad Institute of MIT and Harvard, Cambridge, Massachusetts, 02142, USA; 8 The Dana Farber Cancer Institute, Boston, Massachusetts, 02115, USA; 9 Department of Epidemiology, Harvard School of Public Health, Boston, Massachusetts, 02115, USA; 10 Department of Urology, Örebro University Hospital, Örebro, SE-701 85, Sweden; 11 Harvard Medical School, Boston, Massachusetts 02115, USA; 12 Channing Laboratory, Department of Medicine, Brigham and Women's Hospital, Boston, Massachusetts 02115, USA; 13 Department of Urology, Linköping University Hospital, Linköping, SE 581 85, Sweden; 14 Program in Computational Biology and Bioinformatics, Yale University, New Haven, Connecticut 06520, USA; 15 Department of Computer Science, Yale University, New Haven, Connecticut, 06520, USA; 16 The Howard Hughes Medical Institute at The Broad Institute of MIT and Harvard, Cambridge, Massachusetts, 02142, USA

## Abstract

**Background:**

Current prostate cancer prognostic models are based on pre-treatment prostate specific antigen (PSA) levels, biopsy Gleason score, and clinical staging but in practice are inadequate to accurately predict disease progression. Hence, we sought to develop a molecular panel for prostate cancer progression by reasoning that molecular profiles might further improve current clinical models.

**Methods:**

We analyzed a Swedish Watchful Waiting cohort with up to 30 years of clinical follow up using a novel method for gene expression profiling. This cDNA-mediated annealing, selection, ligation, and extension (DASL) method enabled the use of formalin-fixed paraffin-embedded transurethral resection of prostate (TURP) samples taken at the time of the initial diagnosis. We determined the expression profiles of 6100 genes for 281 men divided in two extreme groups: men who died of prostate cancer and men who survived more than 10 years without metastases (lethals and indolents, respectively). Several statistical and machine learning models using clinical and molecular features were evaluated for their ability to distinguish lethal from indolent cases.

**Results:**

Surprisingly, none of the predictive models using molecular profiles significantly improved over models using clinical variables only. Additional computational analysis confirmed that molecular heterogeneity within both the lethal and indolent classes is widespread in prostate cancer as compared to other types of tumors.

**Conclusions:**

The determination of the molecularly dominant tumor nodule may be limited by sampling at time of initial diagnosis, may not be present at time of initial diagnosis, or may occur as the disease progresses making the development of molecular biomarkers for prostate cancer progression challenging.

## Background

The paramount clinical dilemma in prostate cancer management is how to treat the man with clinically localized disease because the natural history is favorable overall [1] and the benefit from radical treatment modest [2]. Numerous studies have attempted to address this issue but the lack of data with long-term clinical outcomes precludes a definitive assessment. This problem is real and mounting. In 2008, it was estimated that 186,320 new cases of prostate cancer were diagnosed in the United States with the vast majority being clinically localized [3]. The majority of these men are predicted to survive despite prostate cancer for 5 or 10 years regardless of the type of treatment they initially receive [4]. This would suggest that expectant management for localized prostate cancer might be an important modality to deal with this common malignancy. This approach would potentially gain more widespread acceptance if we could sort out those men that were at the greatest risk of disease progression at time of initial diagnosis.

Various approaches using clinical parameters including prostate specific antigen (PSA) levels at time of initial diagnosis have been explored to predict disease progression [5-7]. Although these models work well for men with extreme levels of PSA, the majority of men fall within an intermediate range characterized by a PSA level between 4-10 ng/ml and a Gleason score of 6 or 7. A Gleason score is assigned to a prostate cancer based on its microscopic architectural appearance. It ranges from 2 to 10, with higher values associated with higher tumor grade. The need for additional tests to complement and improve upon these existing approaches would help identify men who must be treated and who can safely be monitored for disease progression.

We reasoned that by performing high-throughput expression profiling of transurethral resection of the prostate (TURP) samples from a large cohort of men on a Watchful Waiting cohort, we would identify a molecular profile predictive of prostate cancer disease progression. We further reasoned that employing a combination of novel technology and a well-defined clinical cohort should yield a strong lethal prostate cancer signature.

Limitations of prior prostate cancer expression profiling studies have included small sample size, restriction of populations to surgical cohorts, short follow up time, and the use of surrogate endpoints such as PSA biochemical recurrence to define disease progression. To overcome these limitations, we designed a study using prostate cancer samples prospectively registered as part of a Watchful Waiting protocol from two regions in Sweden. Up to 30 years of clinical follow up information was available on these men. All of the cases were detected incidentally in a pre-Prostate Specific Antigen (PSA) screening era.

## Methods

### Patient population

The present study is nested in a cohort of men with localized prostate cancer diagnosed in the Örebro (1977 to 1994) and South East (1987 to 1999) Health Care Regions of Sweden. Eligible patients were identified through population-based prostate cancer quality databases maintained in these regions (described in Johansson et al., Aus et al., and Andren et al. [1,8,9]) and included men who were diagnosed with incidental prostate cancer through (TURP) or adenoma enucleation, i.e. stage T1a-b tumors. In accordance with standard treatment protocols at the time, patients with early stage/localized prostate cancer were followed expectantly ("watchful waiting"). No PSA screening programs were in place at the time.

The study cohort was followed for cancer-specific and all cause mortality until March 1, 2006 through record linkages to the essentially complete Swedish Death Register, which provided date of death or migration. Information on causes of death was obtained through a complete review of medical records by a study end-point committee. Deaths were classified as cancer-specific when prostate cancer was the primary cause of death.

We were able to trace tumor tissue specimens from 92% (1256/1367) of all potentially eligible cases. In order to provide complete and consistent information, available hematoxylin and eosin (H&E) slides from each case were reviewed to identify all tissue specimens with tumor tissue. Slides and corresponding paraffin-embedded formalin-fixed blocks were subsequently retrieved and re-reviewed to confirm cancer status and to assess Gleason score and other notable histopathologic features. The reviewers were blinded with regard to disease outcome. Gleason score was evaluated according to Epstein et al. [10]. All patients gave informed consent for the study.

### Study design

**Figure 1:**
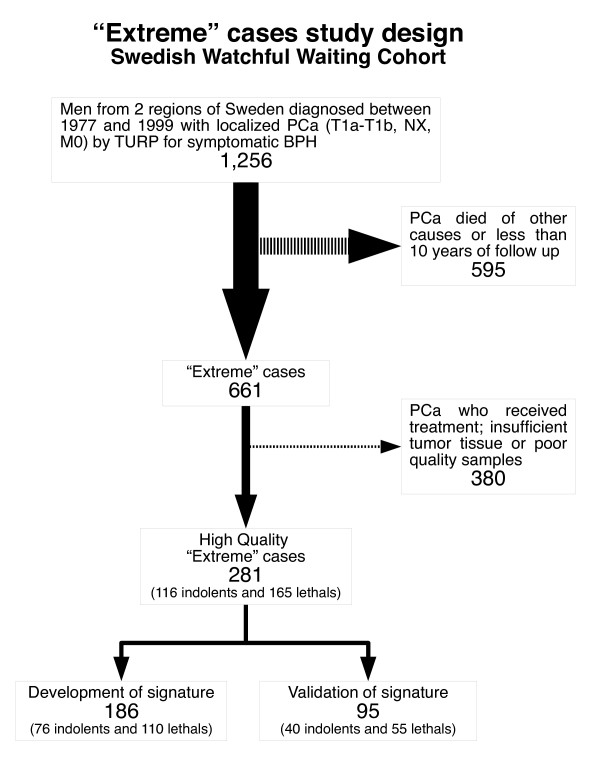
**Study design**. From 1256 men of a Watchful Waiting Cohort, we selected the "Extreme" cases: those who died of prostate cancer or men who lived more than 10 years without signs of progression. We also filtered out some patients based on tumor tissue availability, sample quality or because they were treated. Finally, we randomly divided the patients in a Learning and Validation sets, ensuring that similar proportions of lethals and indolents are present in the two groups.

Since our overarching aim was to identify signatures predicting a lethal or an indolent course of prostate cancer, we maximized efficiency by devising a study design that included men who either died from prostate cancer during follow up (lethal prostate cancer cases) or who survived at least 10 years after their diagnosis (men with indolent prostate cancer). We thus excluded men with non-informative outcomes, namely those who died from other causes within ten years of their prostate cancer diagnosis or had been followed for less than 10 years with no disease progression (n = 595). All men with samples in which high-density tumor regions (defined as more than 90% tumor cells) could be identified were included (n = 381). We excluded from the indolent group men who had received any type of androgen deprivation treatment during follow up (n = 79), since some of these had potentially lethal disease that was deferred by therapy. Twenty-one men were further excluded due to poor sample quality. In total, 281 men (116 with indolent disease and 165 with lethal prostate cancer) were included in the analyses (see Figure 1). The study design was approved by the Ethical Review Boards in Örebro and Linköping. The clinical and pathologic demographics of these of 281 men with prostate cancer are presented in Additional File 1, Table S1.

In addition to the standard pathology evaluation we also characterized each case with respect to ERG gene rearrangement, since it appears that this event is an indicator of poor prognosis (Additional File 1).

### Complementary DNA-Mediated Annealing, Selection, Ligation, and Extension Array Design

An array of 6100 genes (6K DASL) was designed for the discovery of molecular signatures relevant to prostate cancer by using four complementary DNA (cDNA)-mediated annealing, selection, ligation, and extension (DASL) assay panels (DAPs) [11,12]. Details of this procedure can be found in Additional File 1 and also at Gene Expression Omnibus (GEO: http://www.ncbi.nlm.nih.gov/geo/) with platform accession number: GPL5474. This data set is also available at GEO with accession number: GSE16560.

### Supervised classification models: implementation and evaluation

In order to identify and evaluate a predictive molecular signature, six supervised classification models were implemented: k-Nearest Neighbor (kNN) [13], Nearest Template Prediction (NTP) [14], Diagonal Linear Discriminant Analysis (DLDA)[15], Support Vector Machine (SVM)[16], Neural Network (NN)[13], and Logistic Regression (LR)[17]. Their performances were evaluated and compared through a split-sample validation procedure. Specifically, the entire data set was randomly split into a Learning and a Validation sets, with approximately equal proportion of men with lethal and indolent prostate cancer (Figure 1). The Learning set is utilized to create the models and select the best classifier, whose performance is evaluated on the Validation set by means of the Area under the Receiving Operating Curve (AUC). This procedure enables the unbiased estimation of the performance of a classifier since the evaluation is performed on an independent data set [18]. To optimize the classifiers and select the best model, we adopted an iterative cross-validation procedure within the Learning set. The rationale is that results of this procedure enable the identification of the best model which is then used to build a classifier (using the whole Learning set) that is finally evaluated on the Validation set. Specifically, a stratified 10-fold cross-validation split the Learning set in 10 disjoint partitions, *test*_*i *_(*i *= 1..10), with approximately equal proportion of lethal and indolent cases each. Given a partition *test*_*i*_, classifiers were created using the cases not in that partition, i.e. *training*_*i*_, and evaluated on *test*_*i*_. This procedure was repeated 10 times and the final results are averaged across the 10 iterations. Moreover, to avoid potential biases in the selection of the 10 partitions, the entire procedure was repeated 100 times resulting in 1000 different partitions. The best model was then identified by comparing the results obtained on the 100 iterations.

#### Feature Selection

At each iteration of the cross-validation, a feature selection procedure was carried out to identify the subset of genes that are differentially expressed between lethals and indolents. A two-sided t-test was performed for each gene within the *training*_*i *_partition. Different thresholds on the p-values were used for selection (0.01, 0.001). We ensured that the selection of genes is performed using only the samples used for training, avoiding over-fitting the data. For DLDA and the logistic regression models, a stepwise-like feature selection was implemented. Specifically, genes were sorted according to their t-test p-value and then added to the model one at the time. The best gene set is then selected as the one achieving the best AUC with the fewer number of gene predictors.

#### Model selection

Each classifier has its own set of parameters that need to be optimized. The identification of the best parameter set for each classification model was performed within the cross-validation procedure.

### Homogeneity assessment

**Figure 2:**
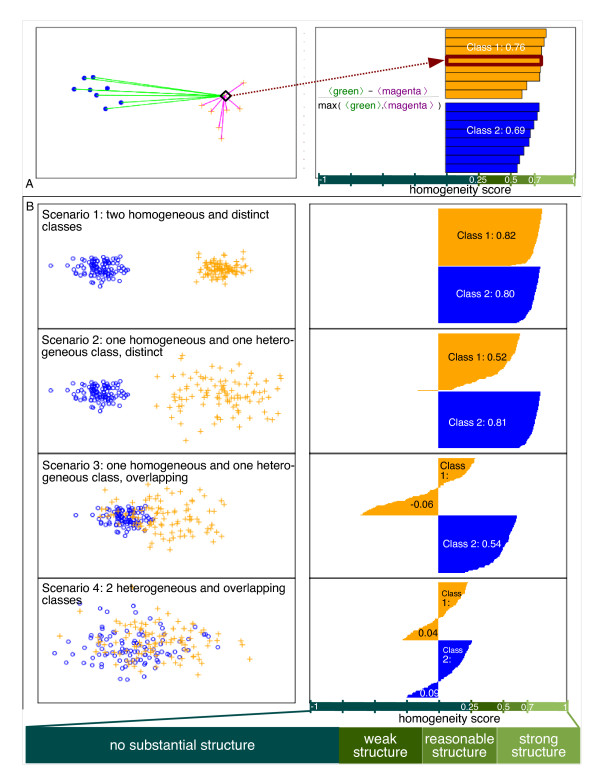
**Schematic of silhouette widths, i.e. homogeneity scores, and silhouette plots**. **A**. (left) Given an element in a group (the orange cross surrounded by a diamond) the distances from elements in the same group (magenta lines) and from those in the other group (green lines) are computed. The homogeneity score can be viewed as the difference between the averages of the inter-group distance (green) and the intra-group distance (magenta). (right) The homogeneity score of each sample is plotted on a horizontal bar, after sorting the samples within each group. The average of the homogeneity scores is computed for each group yielding an estimation of the homogeneity of the cluster. **B**. Four different categories of homogeneity (left) and the corresponding silhouette plots (right) are depicted. Specifically: Scenario 1. two homogeneous and well-separated groups; Scenario 2. one homogeneous and one heterogeneous group, well-separated; Scenario 3. one homogeneous and one heterogeneous group, overlapping; Scenario 4. two heterogeneous overlapping groups. The empirical interpretation of the average homogeneity score for a group is shown at the bottom.

The homogeneity analysis provides an indication of how well samples are clustered into separated groups. Homogeneity is based on the computation of *silhouette widths*, which also enables an intuitive illustration of homogeneity by means of silhouette plots [19]. Briefly, the silhouette width of a sample compares the average distance of that sample from samples of the same group to its average distance from samples of other groups (Figure 2a).

Silhouette widths, here called *homogeneity score*, can be represented through silhouette plots (Figure 2b - right panel). Moreover, the average homogeneity score within a group provides a means to quantify how the samples in the group are similar to each other with respect to the other group: the higher the average homogeneity score is, the more homogeneous the groups is, and the more dissimilar are the elements of this group to the other group (Figure 2b). Details of this analysis are reported in Additional File 1.

We explored for biological heterogeneity (and its converse, homogeneity) in this prostate cancer data set and compared our findings with other tumor tissues. We defined heterogeneity in terms of the molecular signature by evaluating the "distance" between patients belonging to the same group, e.g. lethals, to that of patients belonging to different groups, e.g. indolents. Clearly, in homogeneous tissues, biopsy sampling is not an issue and patients belonging to the same group should be molecularly "closer" to each other than to those belonging to different groups. On the other hand, heterogeneous tissues should not show a clear separation as the molecular profiles of samples in both groups intermingle (Figure 2b - left panel).

We performed the homogeneity analysis on the prostate data set considering the two groups of lethal and indolent patients. Furthermore, we compared these results with 5 well-known publicly available data sets, with different levels of heterogeneity (see Additional File 1 and Additional File 1, Table S2).

## Results

### Association with clinical variables

**Figure 3:**
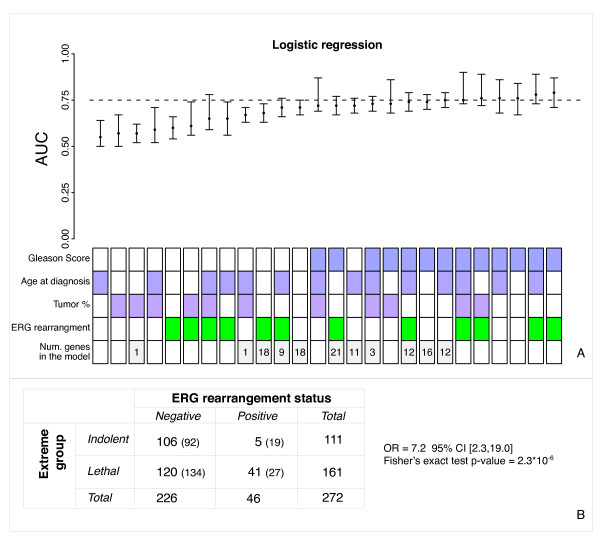
**Supervised analysis**. **A**. Results of logistic regression on the Validation dataset. On top are reported the AUCs of the models, whereas on the bottom the parameters that are used in the corresponding model are shown. A colored square means that the parameter was used in the model, whereas a white square means that the parameter was not used. The last row reports the number of genes that were used by the model, if any. Models including clinical and molecular parameters are reported only if they improved on the corresponding models using clinical parameters only. Models are sorted from left to right according to their AUC. We estimated the Confidence intervals (CIs) for models including genes using the sampling distribution of AUCs generated by the iterative cross-validation procedure on the Learning set. For the other models, a bootstrap estimation of CIs was computed on the Validation set. The genes that are involved in the models are reported in Additional file 1, Table S4. **B**. Contingency table showing ERG rearrangement status association with clinical outcome. In parenthesis the expected numbers of cases if no association is assumed.

We first examined associations between clinical variables and outcome (see Additional File 1, Table S1). Gleason score, divided into 3 groups: 4-6; 7; and 8-10, showed the strongest association with outcome(Cramer's V: 0.45 and Fisher's exact test p-value = 6*10^-14^). In this cohort, men with *ERG *rearranged prostate cancer were significantly more likely to be in the lethal class than the indolent class with an odds ratio of 7.2 (95% CI = [2.8,19.0]; Fisher's exact test p-value = 2.3*10^-6^) (Figure 3).

### Supervised analysis results

The results on the Learning set showed that no classification model clearly outperformed the others in predicting lethal cases (Additional File 1, Table S3). Indeed, most of them had similar performance. Therefore, to simply illustrate and summarize these findings, we report here the complete results of the logistic regression models (Figure 3a).

The molecular classifier alone achieved an AUC of 0.71 (95% CI = [0.67,0.75]) including 18 genes. Surprisingly, however, it did not perform better than models using only clinical features (AUC = 0.76; 95% CI = [0.67,0.84]) for the model with Gleason score). Moreover, when the model combines molecular and clinical features, no improvement over the clinical model was observed (AUC = 0.75; 95% CI = [0.71,0.79]) for the classifier comprising Age, Gleason score and 12 genes.

Gleason score was the most important clinical parameter as all the top models included Gleason score in their classifiers. Although it is well known that inter-observer variability may affect this subjective parameter [20-22], the results demonstrate that it is a strong outcome predictor. Although differences among the top models were marginal, the best classifier of lethal prostate cancer included Gleason score and ERG rearrangement status (AUC = 0.79; 95% CI = [0.71,0.87]).

Lack of a significant improvement in prediction using the molecular profile suggested several possibilities. First, perhaps our definition of lethal and indolent prostate cancer does not capture the biological progression of the tumor. In order to assess how our definition of "extreme" cases affects the results, we ran several experiments by modifying the definition of lethals and indolents. Additional File 1, Table S7 reports the results for DLDA. Similar results are obtained with the other classification models. When the definition of lethal or indolent is very stringent we can achieve some improvement. However, this is obtained at the expense of the number of cases that are classified. Moreover, with very stringent thresholds, we enriched for high and/or low Gleason scores in the two groups. Hence, although a better classification performance can be achieved, it is likely that no additional information about the more critical cases (Gleason score 7) can be obtained. Second, we reasoned that stroma-contaminated samples may have prevented us to discover a molecular signature of aggressive prostate cancer. Therefore, in order to seek for stroma-contaminated samples, we employed a molecular profile developed by Tomlins et al. [23] where they applied laser capture micro-dissection (LCM) to prostate tissues (see Additional File 1 for details). We identified in our data set a cluster of samples exhibiting stroma-like profile based on a set of 47 top ranked common genes (see Additional File 2, Figure S3). These samples (n = 17) were then excluded from the Learning set and the remaining samples were used as a new Learning set. The same iterative cross-validation procedure was employed for a SVM classifier (polynomial degree = 1; cost = 0.1; p-value = 0.01) which achieved an AUC of 0.77 (95%C.I. [0.73-0.81]). We believe that this result, which is comparable to the one using the full set (see Additional File 1, Table S3), is not sufficient to argue that stroma-contaminated tissue have prevented us to develop an accurate prediction model. Furthermore, we considered that, perhaps, the genes assayed on the this DASL array platform might not include the actual genes driving tumor progression. However, the 6K DASL gene set was developed specifically for this project. We selected genes showing the maximum variation in expression in 24 expression profiling studies from 15 different tumor types or because they were transcriptionally deregulated in previous prostate cancer studies. These genes cover most of the known pathways. Moreover, we demonstrated that this same platform and a slightly larger cohort can reliably identify a molecular signature for *ERG *rearrangement status [24]. Nevertheless, we performed an additional analysis by evaluating the consistency of the Gleason-score correlated genes (see Additional File 1) confirming its reliability. We thus favored that inter-tumor heterogeneity was the main reason and thus explored the potential impact of tissue heterogeneity by performing a homogeneity analysis.

### Homogeneity analysis results

For prostate cancer, we computed homogeneity scores of the samples using a subset of the genes assessed on the array. We selected the genes that best distinguish the two groups, namely lethal and indolent prostate cancer, on the entire cohort of 281 patients, intentionally over-fitting the data to obtain the best molecular descriptors of the two groups. Specifically, genes were selected by a two-tailed t-test p-values after correcting for multiple hypothesis testing (q-value < 0.05), yielding 118 genes (see Additional File 1, Table S5)[25].

**Figure 4:**
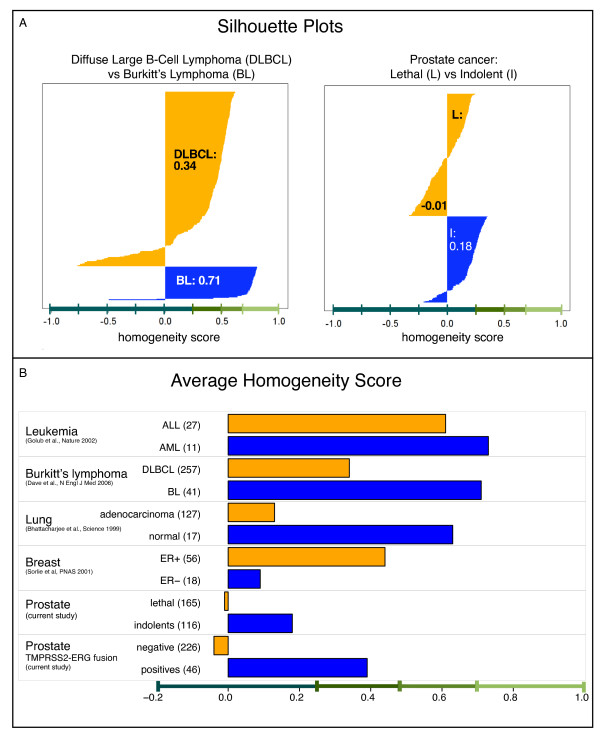
**Homogeneity analysis**. **A**. Silhouette plot for Burkitt's lymphoma (left) and prostate cancer (right). The numbers report the average homogeneity score for each group. **B**. Average homogeneity score for different cancer data sets.

We performed the same analysis for other tumor data sets and compared the results with our data set. For illustration purposes, Figure 4a shows the silhouette plot for our prostate cancer data set compared with the Burkitt's lymphoma data set [26]; whereas Figure 4b reports the results for all data sets. We compared prostate cancer with Burkitt's lymphoma because both harbor a recurrent translocation that leads to the over expression of two known oncogenes: c-MYC for Burkitt's lymphoma and ERG for prostate cancer.

The results support the heterogeneity hypothesis for prostate cancer. The average homogeneity score of the lethal group is lower than zero, meaning that on average, samples in the lethal group are more similar to samples in the indolent group. On the other hand, indolent cases seem to be slightly more homogeneous than lethal, as expected, although the average homogeneity score is rather low.

Conversely, the homogeneity scores on Burkitt's lymphoma data set are quite striking when compared with prostate cancer. Burkitt's lymphoma is a molecularly defined disease, with marked differences with respect to the broader class of lymphoma. Dave et al. identified a signature comprising 228 genes which is able to discriminate between Diffuse Large B-Cell Lymphoma (DLBCL) and Burkitt's lymphoma. This signature resulted in an average homogeneity score of 0.71, suggesting a *strong structure *of Burkitt's lymphoma. This is in contrast with the DLBCL group, which is more heterogeneous and consists of multiple sub-classes. The homogeneity analysis confirms this notion yielding an average homogeneity score of 0.34, interpreted as a *weak structure *(see Additional File 1 for additional detail).

Among the other studies, AML and ALL show the highest degree of homogeneity with both classes scoring higher than 0.6, whereas breast and lung cancer are confirmed to be heterogeneous (Figure 4b). Similarly to prostate cancer, we selected the most informative genes separating the groups for each study. Specifically, the most informative genes of Sørlie et al. [27] were selected by computing a Wilcoxon test between ER+ and ER- samples and using a p-value cut-off of 0.01. Battacharjee et al. [28] identified 675 genes whose differential expression levels were the most highly reproducible. For the leukemia data set, we selected to top 50 genes according to the correlation-based score proposed by Golub et al. [29](see Additional File 1 for more detail).

### Homogeneity of ERG rearranged subclass

We recently reported a molecular signature including 87 genes characteristic of *ERG *rearranged cases in the same cohort of patients [24], which was also validated on a U.S. based cohort. The homogeneity analysis using this gene signature supports the hypothesis that *ERG *rearranged cases represent a distinct subclass, although we cannot extend this result for the entire population of *ERG *rearranged prostate cancers. Indeed, these cases show a homogeneity score of 0.39 (Additional File 2, Figure S2).

## Discussion

Current prognostic models of prostate cancer, including PSA, Gleason score and clinical stage fail to accurately predict disease progression, especially for men with intermediate disease. Two large randomized trials evaluating the effect of PSA screening on prostate-cancer mortality, namely the Prostate, Lung, Colorectal, and Ovarian (PLCO) and the European Randomized Study of Screening for Prostate Cancer (ERSPC), showed that during the first decade of follow-up, PSA screening has at best a modest effect (20% relative reduction of PCA specific death in the ERSPC) on PCA mortality, with substantial risks of negative biopsy, over diagnosis and over-treatment [30,31]. The need to better identify patients with a more aggressive disease is thus an open challenge given the clinical observation that prostate cancer is a heterogeneous disease. This observation is based on the experience of clinicians who witness men with localized disease that should fair well but on occasion do not and less commonly men with apparently aggressive disease who do well. How can we account for this clinical heterogeneity? We anticipated that a well-designed molecular study interrogating thousands of genes implicated in cancer and specifically prostate cancer would help us determine a molecular signature for lethal and indolent disease. Perhaps what is clinically referred to as "heterogeneity" really represents our inability through Gleason grading or other clinical attributes to untangle the key elements that would, if known, help us predict which men will succumb to disease progression. The findings of the current study and other recent studies described below point to a more concerning reality about what accounts for heterogeneity.

This study found that molecular predictors can distinguish aggressive from indolent prostate cancer similarly to models generated from Gleason score and other clinical parameters. However, by combining clinical and molecular data, we were not able to improve on known predictors. The explanation is manifold. First, we must consider the important limitation of prostate cancer sampling. We know that a prostate gland harbors often up to 5 geographically distinct tumor nodules [32-36] and these nodules are often clonally distinct. If we consider the homogeneity of *ERG *rearrangement in circulating tumor cells (CTCs) [37] and that the ETS gene rearrangements occur early in the development of prostate cancer, as they are often seen in high-grade PIN [38], and, when observed, are present in all tumor cell within a nodule; then, we can consider this a possible marker of tumor clonality. Observations from three independent groups demonstrate that up to 50% of prostate cancers with multiple nodules have clonally distinct lesions [39-41]. This would strongly support why sampling of the "right" cancerous nodules is so critical in prostate cancer. A prostate needle biopsy or TURP sample may or may not capture the driving lesion leaving an important clone undetected. This inability to identify the molecularly dominant nodule (intra-tumor heterogeneity) would then help explain the "heterogeneity" observed in the clinical assessment at time of diagnosis with outcome.

However, if intra-tumor heterogeneity were the main explanation for our results, and inter-tumor heterogeneity, i.e. the presence of many alternative pathways which lead to lethality in prostate cancer, only marginal, then all cancer foci across individuals should share a similar molecular profile. How does sampling play a role in this scenario? The set of indolent prostate cancer samples is not affected by sampling, the set of lethal prostate cancer samples is affected, in that the lethal focus is 'sub-sampled'. Let assume that this causes a 50% dilution of the lethal molecular signal. Due to our study design and combinations of supervised and unsupervised analysis approaches, we should still have been able to detect the presence of a strong and consistent lethal signal, even if this was for a subset of the lethal prostate cancer population. Hence, we believe that our results are best explained by high degree of heterogeneity between lethal prostate cancers.

However, another possible alternative explanation for clinical heterogeneity might be that the lethal signature develops with the accumulation of molecular lesions over time and therefore may not be present at time of initial diagnosis in contrast to the homogeneity of *ERG *rearrangement in CTCs [37]. This would not be mutually exclusive from inter-tumor heterogeneity but could compound the problem. Finally, the molecular signature may be embedded in the adjacent non-cancerous stromal tissue as recently observed in hepatocellular cancer [42] or perhaps due to a host immune response to the tumor that might not be measurable by examination of the tumor sample. Regardless of what the mechanism or combination of mechanisms is, we are still faced with an inability to consistently detect the lethal molecular signature as observed in the current study.

Our study results are in fact consistent with other emerging data from U.S. cohorts using similar and different molecular platforms. Nakagawa et al. recently attempted to develop a biomarker panel to predict which men with rising PSA following surgery would progress with clinically significant disease [43]. They employed a case-control design where cases were defined as men with rising PSA who progressed within 5 years after initial surgery. Controls were men with rising PSA but no sign of clinical disease progression within the first 5 years following surgery. A total of 213 cases and 213 controls were used for this study and, similar to the current study, the cases and controls were divided into training and validation set. Although the results on the training set seemed promising (see Additional File 1), the validation phase showed mis-classifications in both directions and none of the models with molecular and clinical parameters performed better than an AUC of 0.75 [43].

Another recent study is significant because a two-phase biomarker development approach was used to classify long-term disease progression or death due to prostate cancer. Cheville et al. reported on a molecular classifier developed using a profile developed from tumor samples isolated by laser capture micro-dissection [44]. They used quantitative RT-PCR to measure gene expression and cancer specific death following surgery or development of metastatic disease as the clinical endpoint. They used a 2-phase design with a training set of 157 high-risk patients and a validation set of 57 high-risk patients. Their results demonstrated that a model including topoisomerase-2a, cadherin-10, ETS genes involved in gene fusion (i.e., ERG, ETV1, and ETV4), and aneuploidy status had an AUC of 0.81 and 0.79 for training and validation sets, respectively.

Based on the published series (Nakagawa et al., Glinsky et al., Lapointe et al., Singh et al., Yu et al., Cheville et al.) and the current study, it is therefore impressive that all of these reports using different platforms and patient populations achieve similar results [43-49].

Although other explanations may be possible, we favor that inter-tumor heterogeneity plays a more critical role. The strongest evidence from the current study has to do with the association of *ERG *rearrangement status and lethality (see also Attard et al. [37]).

The association between *ERG *rearranged cases and the lethal phenotype suggests that *ETS *rearrangements describe a particularly aggressive subclass of prostate cancer. In the current study 41 of 46 ERG rearranged prostate cancers were lethal; the unadjusted odds ratio for lethal disease associated with ERG rearrangement status was 7.2 (95% CI 2.8-19.0). This confirms and extends observations from 111 men in the expectant management cohort from Örebro where men with ERG rearranged prostate cancer were significantly more likely to have lethal disease than men with fusion negative tumors (cumulative incidence ratio = 2.7, p-value < 0.01, 95% CI = [1.3,5.8]) [50]. From the United Kingdom, Attard et al. reported associations between *TMPRSS2-ERG *fusion with interstitial deletion and cause specific survival taking into account age, Gleason score, and pre-treatment PSA in a cohort of 445 men conservatively treated for prostate cancer [51]. Interestingly, aneuploidy in combination with *TMPRSS2-ERG *fusion was associated with the worst clinical outcome (hazard ratio = 6.10, 95% CI = [3.33,11.15], p-value < 0.001, 25% survival at 8 years). The relatively low frequency of *ERG *rearrangement in this cohort may represent the admixture of peripheral zone tumors with a presumed *ERG *rearrangement frequency of 45% [52] and transition zone tumors with a significantly lower *ERG *rearrangement frequency [53].

## Conclusions

In summary, this study attempted to identify a molecular signature for lethal prostate cancer. Molecular profiles developed in this study performed similar to clinical models and no model was identified that improved on the clinical models by including the profiling data. One significant result is the association of *ERG *rearrangement with lethality (OR = 7.2 95% CI = [2.3,19.0], Fisher's exact test p-value = 2.3*10^-6^). Although other explanations may be plausible, we believe that prostate cancer tumor heterogeneity is highly likely to be a major limitation in the development of a lethal prostate cancer signature.

This study underlines the importance of developing a better strategy to best capture the molecular complexity of prostate cancer. One possibility could be using circulating tumor cells, known as liquid biopsies, to reduce the confounding effect of sampling multiple tumor nodules in a prostate gland and improve the current biopsy strategy [54,55]. After which, we might be able to focus on characterizing the multiple lethal signatures that may exist.

## Competing interests

The authors declare that they have no competing interests.

## Authors' contributions

ASB carried out the supervised analysis related to SVM, NN and logistic regression; conceived the homogeneity analysis and implemented the silhouette widths and plots; and drafted the manuscript. FD participated in the design of the supervised analysis and carried out the SVM and NN analysis. SC and YP participated in the design of the supervised analysis and carried out the DLDA and logistic regression. YP also coordinated the supervised analysis. SRS characterized the *ERG *rearrangement status of the samples and helped to draft the manuscript. YH participated in the generation of the expression measurements and carried out the supervised analysis with NTP and k-NN. SP evaluated the pathology specimen of all the samples, participated in the characterization of *ERG *rearrangements and help to draft the manuscript. HOA participated in the design of the study and its coordination. KF helped to organize the selection of the patients and to draft the manuscript. LAM participated in the statistical analysis of the results. PWK participated in the design and coordination of the study. MS participated in the design of the study and helped the statistical analysis of the results. SOA, EV, JEJ participated in the design of the study, collected and curated the samples. MBG participated in the supervised and the homogeneity analysis and helped to draft the manuscript. TRG participated in the design of the study and helped coordinating the gene expression measurements. MAR participated in the study design and in its coordination, evaluated the pathology specimen, helped to draft the manuscript. OA participated in the study design and its coordination.

All authors read and approved the final manuscript.

## Pre-publication history

The pre-publication history for this paper can be accessed here:

http://www.biomedcentral.com/1755-8794/3/8/prepub

## Supplementary Material

Additional file 1 **Supplementary material**. This file contains additional information regarding the experimental protocols, the supervised data analysis and the homogeneity data analysis, as well as additional results to further support the main conclusions. Click here for file

Additional file 2 **Supplementary figures**. This file contains supplementary figures. Click here for file
