# Supplementary material for: Molecular sampling of prostate cancer: a dilemma for predicting disease progression
Source: BMC Med Genomics. 2010 Mar 16;3:8. doi: 10.1186/1755-8794-3-8 (PMC2855514; doi:10.1186/1755-8794-3-8)
Supplement: Additional file 1 — Supplementary material. This file contains additional information regarding the experimental protocols, the supervised data analysis and the homogeneity data analysis, as well as additional results to further support the main conclusions. [file 1755-8794-3-8-S1.DOC]

**Supplementary material of:**

Molecular Sampling of Prostate Cancer: a dilemma for predicting disease progression

Andrea Sboner^1^, Francesca Demichelis^2,3^, Stefano Calza^4,5^, Yudi Pawitan^4^, Sunita R Setlur^6^, Yujin Hoshida^7,8^, Sven Perner^2^, Hans-Olov Adami^4,9^, Katja Fall^4,9^, Lorelei A Mucci^9,11,12^, Philip W Kantoff^8,11^, Meir Stampfer^9,11,12^, Swen-Olof Andersson^10^, Eberhard Varenhorst^13^, Jan-Erik Johansson^10^, Mark B Gerstein^1,14,15^, Todd R Golub^7,8,16,#^, Mark A Rubin^2,7^,*^,#^, Ove Andrén^10,#^

^1^ Department of Molecular Biophysics and Biochemistry, Yale University, New Haven, Connecticut, 06520, USA.

^2^ Department of Pathology and Laboratory Medicine, Weill Cornell Medical Center, New York, New York, USA.

^3^ Institute for Computational Biomedicine, Weill Cornell Medical Center, New York, New York, USA.

^4^ Department of Medical Epidemiology and Biostatistics, Karolinska Institutet, Stockholm, Sweden.

^5^ Department of Biomedical Sciences and Biotechnologies, University of Brescia, Brescia, Italy.

^6^ Department of Pathology, Brigham and Women’s Hospital, Boston, Massachusetts, 02115, USA.

^7^ The Broad Institute of MIT and Harvard, Cambridge, Massachusetts, 02142, USA.

^8^ The Dana Farber Cancer Institute, Boston, Massachusetts, 02115, USA.

^9^ Department of Epidemiology, Harvard School of Public Health, Boston, Massachusetts, 02115, USA.

^10^ Department of Urology, Örebro University Hospital, Örebro, SE-701 85, Sweden.

^11^ Harvard Medical School, Boston, Massachusetts 02115, USA.

^12^ Channing Laboratory, Department of Medicine, Brigham and Women’s Hospital, Boston, Massachusetts 02115, USA.

^13^ Department of Urology, Linköping University Hospital, Linköping, SE 581 85, Sweden.

^14^  Program in Computational Biology and Bioinformatics, Yale University, New Haven, Connecticut 06520, USA

^15^ Department of Computer Science, Yale University, New Haven, Connecticut, 06520, USA

^16^ The Howard Hughes Medical Institute at The Broad Institute of MIT and Harvard, Cambridge, Massachusetts, 02142, USA.

^#^ These authors share senior authorship.

# Methods

## Complementary DNA–Mediated Annealing, Selection, Ligation, and Extension Array Design Gene expression arrays

We designed four complementary DNA (cDNA)–mediated annealing, selection, ligation, and extension (DASL) assay panels (DAPs) for the discovery of molecular signatures relevant to prostate cancer [1, 2]. An extensive analysis of previously generated microarray datasets, including 24 studies, 2149 samples, and 15 tissue types allowed us to prioritized informative genes, that is, genes showing the largest variation in expression across samples (the datasets are available at http://www.broad.mit.edu/cancer/pub/HCC). The top-ranked transcriptionally informative genes comprised genes in most of the known biological pathways. Details of this procedure can be found in Hoshida et al. [3]. Furthermore, to ensure that prostate cancer–related genes were included in the DASL assay panel, a meta-analysis of previous microarray datasets from the Oncomine database was carried out [4-7]. Genes that were transcriptionally regulated in prostate cancer in that list were also included. The final array consisted of 6100 genes (6K DAP). Quality assessment was performed by exploiting the negative control probes present on the array. Assuming a log-normal distribution of negative controls, we computed the mean and standard deviation within each DAP. We then compared each gene measurement with this negative control distribution. An observed value is considered “present” if the probability of being greater than negative controls is greater than 95%. For each sample we compute the proportion of “present” genes. Based on a comparison with the “median” array, i.e. the array constructed by computing the median for each gene, we excluded poor quality samples with less than 55% valid measurements. Finally, the remaining raw data were normalized using the cubic spline algorithm. This procedure has been adapted from Hoshida et al. [3].

## Sample Processing and Complementary DNA–Mediated Annealing, Selection, Ligation, and Extension

Details of sample processing were reported in Setlur et al. [8]. Briefly, 0.6mm biopsy cores were taken from tumor-enriched areas (>90% tumor) of formalin fixed paraffin embedded tissue blocks. RNA was extracted from these cores in a 96-well format using the CyBi-Well liquid handling system (CyBio AG, Jenna, Germany). The cores were first deparaffinized and the RNA was extraction using TRIzol LS reagent. The RNA was quantified using a Nanodrop spectrophotometer (NanoDrop Technologies, Wilmington, DE). 400ng RNA was used for the DASL assay.

## ERG rearrangement status determination

A recurrent chromosomal aberration involving the rearrangement of the 5′-untranslated region of the androgen-regulated transmembrane protease serine 2 (*TMPRSS2*) promoter with erythroblast transformation–specific transcription factor family members has been found in the majority of prostate cancers among several PSA-screened populations [9]. The common fusion between *TMPRSS2* and v-ets erythroblastosis virus E26 oncogene homolog (avian) (*ERG*) is associated with a more aggressive clinical phenotype, implying the existence of a distinct subclass of prostate cancer defined by this fusion [10]. We employed an ERG break-apart fluorescence in situ hybridization (FISH) assay to determine *ERG* rearrangement status. If cases were not assessable by FISH, qPCR was used for a total of 78 cases [11]. An aliquot of the RNA used for DASL was used for qPCR. cDNA was synthesized as above using the Illumina kit (Illumina Inc). The *TMPRSS2–ERG* fusion product was detected using SYBR green assay (QIAGEN Inc) with *TMPRSS2–ERG_f* and *TMPRSS2–ERG_r* primers (GenBank accession code NM_ DQ204772.1)[9]. RPL13A was used for normalization. We used RNA from NCI-H660 cells, which express *TMPRSS2–ERG* [12] as a positive control and a calibrator for quantification. Relative quantification was carried out using the comparative ΔΔC_t_ method [13].

## Supervised classifications: finding the best classification model

In order to identify the best classification model, we tested several algorithms on a subset of prostate cancer cases. The entire dataset was randomly split into a Learning and a Validation sets, with approximately equal proportion of men with lethal and indolent prostate cancer (see the main text for a definition of indolent and lethal cases). The Learning set included 186 men (76 indolent and 110 lethal cases), whereas the Validation set comprised 95 men (40 indolent and 55 lethal cases). The rationale was to apply the best model selected on the Learning set to the Validation set for final performance evaluation. Six different classification algorithms were employed:

a) k-Nearest Neighbor (kNN), (k in 3:11) [14];

b) Nearest Template Prediction (NTP) [15];

c) Diagonal Linear Discriminant Analysis (DLDA) [16];

d) Support Vector Machine (SVM) with polynomial and radial basis kernels (degree in 1:3, cost in 10^(-2:1), gamma in 10^(-4:-2)) [17];

e) Neural Networks (NN), (decay in 10^(-3,-1), size in {3,5,7}) [14];

f) Logistic Regression (LR) [18].

The dependent variable in the classification problem is the extreme status, either *Indolent* or *Lethal*. Hereafter we describe the analysis that was performed on the Training set to select the best classification model.

**Analysis Schema:** We employed a 10-fold cross validation with 100 random replication (unless differently specified) of the 10-fold split. The folds are balanced for extreme status and follow up time.

*Performance estimation:* Performance of classifiers was evaluated by computing the Area under the Receiver Operating Curve (AUC), a measure that accounts for the imbalance between the classes. This iterative evaluation framework also enables the estimation of the confidence intervals of the AUC by computing the standard error of the sampling distribution for the models including molecular features.

**Feature Selection:** At each iteration of the cross-validation, a feature selection procedure was carried out to identify the subset of genes that are differentially expressed between lethals and indolents. A two-sided t-test was performed for each gene within the *training_i_* partition. Different thresholds on the p-values were used for selection (0.01, 0.001). We ensured that the selection of genes is performed using only the samples used for training, avoiding over-fitting the data. For DLDA and the logistic regression models, a stepwise-like feature selection was implemented. Genes were sorted according to their t-test p-value and added to the model one at the time. The best model is then selected as the one achieving the best AUC with the fewer number of gene predictors.

**Model selection:** Each classifier has its own set of parameters that needs to be optimized. The identification of the best parameter set for each classification model was performed within the cross-validation procedure. As an example, Support Vector Machines (SVMs) require a misclassification cost to be specified as well as a kernel function. Hence, many SVMs were created, each with a different set of parameters. The results obtained by the cross-validation procedure on the Training set were used to select the optimal set of parameters. **Additional File 1, Table S3** reports the results of the different algorithms on the Learning set. Only the models with the optimal parameter set are reported.

**Class randomization**

To assess the reliability of the signal we detect, we also randomized the class labels for the samples and ran the classification algorithms. We expect an average of 50% error rate across the number of optimal predictors. Indeed, the results show an average of 50%, ranging between 46%-57% for kNN, 41%-61% for DLDA and 43%-57% for SVM.

## Homogeneity Score

The homogeneity score is based on the computation of silhouette widths [19]. Formally, given a dissimilarity metric *d*, a silhouette width *s_i_* can be defined for each sample *i* as follows:

where *a_i_* is the average dissimilarity of sample *i* from all other samples in the same group; *b_i_* is the average dissimilarity of sample *i* from samples in the other group (**Figure 2a**)[[1]](#footnote-2). According to this definition *s_i_* ranges from -1 to 1. This provides a straightforward interpretation of this measurement. Three defining situations can be described:

1. when *s_i_* is close to 1, *b_i_* is much greater than *a_i_*. This means that the average dissimilarity of sample *i* from samples belonging to a different group is higher than that from samples of the same group. Hence, sample *i* is well defined and there are no doubts about its classification.
2. when *s_i_* is close to -1, *a_i_* is much greater than *b_i_*, thus sample *i* is closer to the samples of the other group than to those in the same group. It is very likely that sample *i* has been misclassified;
3. when *s_i_* is zero, sample *i* is, on average, equally distant from all samples and thus it can belong to either group. In other words, it is not clear which group sample *i* belongs to.

The silhouette width is therefore a straightforward quantitative measurement of homogeneity. Hereafter, silhouette width is thus called *homogeneity score*. Silhouette plots can provide an intuitive visualization of homogeneity scores. In a silhouette plot samples are sorted within each group according to their homogeneity score and then plotted as horizontal bars (**Figure 2b** – right panel). If partitioning of samples results in homogeneous groups, the majority of homogeneity scores will be close to 1. Therefore the bars in a silhouette plots will lean towards the right side. Conversely, a heterogeneous group will have several samples with zero or negative homogeneity scores that can be easily identified from the plot. For this reason, silhouette plots provide also a means of identifying mislabeled samples.

Furthermore, the silhouette widths of a group *G* can be summarized by computing their average within that group:

*S_G_* can be seen as a measure of homogeneity of an entire group, being close to 1 if many samples of the group have high homogeneity scores. Indeed, in terms of group structure, the usual interpretation of average homogeneity scores *S_G_* is: i. *S_G_ ≤* 0.25 *no structure*; ii. 0.25 < *S_G_ ≤* 0.50 *weak structure*; iii. 0.50 < *S_G_ ≤* 0.70 *reasonable structure*; and iv. *S_G_* > 0.70 *strong structure*.

The dissimilarity between two samples *i* and *j* can be defined in many ways. In our case, it is based on the Pearson's correlation of their expression profiles: *d*(*i,j*) = 1 – *corr*(*i,j*). Intuitively, if the expression profile of two samples correlates then their dissimilarity should be close to zero. Conversely, two uncorrelated samples will have high dissimilarity.

## Homogeneity analysis of Burkitt's Lymphoma subclasses

We performed the homogeneity analysis considering the sub-classes of Diffuse Large B-Cell Lymphoma (DLBCL). As expected, each sub-class of DLBCL is more homogeneous. Indeed, by carrying out the same analysis comparing BLs with Activated B-cell-like (ABC), one of DLBCL subclasses, we obtained an average homogeneity score greater than 0.50 for the ABC group, suggesting *reasonable* to *strong structure* (**Additional File 2, Figure S1**). Similar results were obtained for the other sub-classes of DLBCL (**Additional File 2, Figure S1**).

## Data sets for homogeneity analysis

The data sets used for the homogeneity analysis are summarized in **Additional File 1, Table S2**. A breast cancer data set is that by Sørlie et al. including 85 samples, out of which 56 are ER positive [20]. Genes differentially expressed between ER+ and ER- samples were selected by means of Wilcoxon test with a p-value cut-off of 0.01. Bhattacharjee et al. dataset (Dataset_B) compares 127 lung adenocarcinomas with 17 normal lung tissue [21]. Six-hundred seventy-five genes are here used for the homogeneity analysis which were identified as the transcripts whose expression levels were the most highly reproducible. Golub et al. dataset explores Acute Myeloid Leukemia (AML: 11 samples) and Acute Lymphoblastic Leukemia (ALL: 27 samples) and shows that the two groups have rather distinct molecular profiles [22]. This dataset is available from the package “golubEsets” in Bioconductor package repository for R [23]. We here selected to top 50 genes according to the correlation-based score proposed by the authors [22]. Finally, we employed a Burkitt's lymphoma dataset including 303 cases [24]. This study shows that Burkitt's lymphoma is a well-characterized subclass of lymphomas and has a peculiar gene expression profile compared with diffuse large B-cell lymphoma (DLBCL) including 228 genes.

## Search for possibly stroma contaminated sample.

We reasoned that stroma contaminated samples may have prevented us to discover a molecular signature of aggressive prostate cancer. Therefore, in order to seek for stroma contaminated samples, we employed a molecular profile developed by Tomlins et al. [25] where they applied laser capture microdissection (LCM) to prostate tissues. mRNA expression was then assessed with an Affymetrix platform. The dataset includes 12 stromal cell samples and 30 PCa. Focusing on this subset, we selected genes able to distinguish between stromal cells from PCa. The initial set includes ~ 9.100 genes; genes with less than 50% of presence call were excluded. A P-value cut off was set at 10^-6^ (0.01/10.000 - Bonferroni-like correction). Two-hundred eighty-seven (287) genes passed the filter (the top up and down genes are reported in **Additional File 2, Figure S3a**). We identified in our dataset a cluster of samples exhibiting stroma-like profile based on a set of 47 top ranked common genes (see **Additional File 2, Figure S3b**). These samples (17) were then excluded from the training set. The remaining samples were used as a new training set and the same iterative cross-validation procedure was employed for a SVM classifier (polynomial degree=1; cost=0.1; p-value=0.01). The SVM achieved an AUC of 0.77 (95%C.I. [0.73-0.81]). This result is comparable to the one using the full set (see **Additional File 1, Table S3**), thus not sufficient to argue that stroma contaminated tissues play a major role in preventing the development of an accurate prediction model.

## Assessment of the platform: Gleason score-correlated genes

Gleason score is assumed to have strong association with molecular characteristic of prostate cancer. We aimed to assess the validity of our dataset generated using formaline-fixed paraffin-embedded (FFPE) tissues by evaluating the consistency of the expression pattern of Gleason score-correlated genes with published microarray dataset generated by standard microarray using intact RNA [26].

The Swedish cohort includes cases with relatively higher Gleason score. We have information about major and minor Gleason score for the Swedish dataset only (**Additional File 1, Table S6**). Note that here we report the information available for a larger dataset, including the patients that were excluded because of hormone treatment. Since Gleason score is evaluated on biopsies at the time of diagnosis, it is independent from subsequent treatment.

In the Swedish dataset, Gleason score-correlated genes were selected as (1) differentially expressed genes between major Gleason “3” and “4 and greater (4+)” by t-test, and (2) genes with higher Pearson correlation coefficient. The significance was assessed by random permutation test by scrambling the sample labels (n=1000). A false discovery rate (FDR) of 0.05 was used as the significance threshold. Genes in Singh dataset were rank-ordered according to the Pearson correlation coefficient between log2-transformed gene expression data and the Gleason score.

The over-representation of the Swedish Gleason-correlated genes in either of the positive or negative correlation side of the Singh’s ranked gene list was evaluated by Gene Set Enrichment Analysis (GSEA) [27].

**(1) Differentially expressed genes between major Gleason “3” and “4 and above”**

By the permutation t-test, 298 over-expressed and 245 under-expressed genes in the “4+” group in the Swedish cohort passed the significance threshold. In GSEA, the over-expressed genes were significantly over-represented in the “positive correlation” side (FDR q= 0.002), and the under-expressed genes tended to be over-represented in the “negative correlation” side in Singh's dataset (FDR q=0.054) (**Additional File 2, Figure S4a**).

**(2) Genes with higher Pearson correlation coefficient**

By the Pearson correlation coefficient computation in the Swedish data set, 203 positively and 101 negatively correlated genes passed the significance threshold. Among them, 144 and 78 genes were common to the genes over- and under-expressed genes in the “4+” group, respectively. In the GSEA, the positively correlated genes were significantly over-represented in the “positive correlation” side (FDR q= 0.021), and the negatively correlated genes were significantly over-represented in the “negative correlation” side in Singh dataset (FDR q=0.006)(**Additional File 2, Figure S4b**).

Hence, we conclude that our data set shows consistent expression of Gleason-correlated genes with published dataset. Furthermore, since Gleason score is a subjective parameter [28-30], with this analysis we could also conclude that inter-observer variability does not affect the evaluation of the molecular features.

## Limited improvement from classification models including molecular features

We show in the main paper that the other studies have similar results. In particular, here we report some additional details about the study of Nagakawa et al.[31]. In addition to the standard commercially available Illumina DASL expression microarray (CancerPanel^TM^ v1), they used a custom DASL platform containing 526 genes, including other biomarkers from previous prostate cancer biomarker studies. The final testing model included 570 prostate cancer related genes and 451 other cancer-related genes.

In their training sets, models using only clinical parameters demonstrated AUC ranging from 0.74-0.78 whereas their best molecular classifier including 17 genes had an AUC of 0.85 (95% CI=[0.81-0.90]). This classifier was improved by including the clinical parameters producing AUCs ranging from 0.86-0.88. They also applied previously published clinical models to their data set with and without their gene model [26, 32-35]. Although the results on the training set seemed promising, the validation phase showed mis-classifications in both directions and none of the models including the 17-gene model with clinical parameters performed better than an AUC of 0.75 [31].

# References

1. Fan J, Yeakley JM, Bibikova M, et al.: **A Versatile Assay for High-Throughput Gene Expression Profiling on Universal Array Matrices**. *Genome Res.* 2004, **14**:878-885.

2. Bibikova M, Talantov D, Chudin E, et al.: **Quantitative Gene Expression Profiling in Formalin-Fixed, Paraffin-Embedded Tissues Using Universal Bead Arrays**. *Am J Pathol* 2004, **165**:1799-1807.

3. Hoshida Y, Villanueva A, Kobayashi M, et al.: **Gene Expression in Fixed Tissues and Outcome in Hepatocellular Carcinoma**. *N Engl J Med* 2008, **359**:1995-2004.

4. Rhodes DR, Yu J, Shanker K, et al.: **ONCOMINE: a cancer microarray database and integrated data-mining platform**. *Neoplasia* 2004, **6**:1-6.

5. Rhodes DR, Kalyana-Sundaram S, Mahavisno V, et al.: **Mining for regulatory programs in the cancer transcriptome**. *Nat Genet* 2005, **37**:579-83.

6. Rhodes DR, Kalyana-Sundaram S, Mahavisno V, et al.: **Oncomine 3.0: genes, pathways, and networks in a collection of 18,000 cancer gene expression profiles**. *Neoplasia* 2007, **9**:166-80.

7. Rhodes DR, Kalyana-Sundaram S, Tomlins SA, et al.: **Molecular concepts analysis links tumors, pathways, mechanisms, and drugs**. *Neoplasia* 2007, **9**:443-54.

8. Setlur SR, Mertz KD, Hoshida Y, et al.: **Estrogen-Dependent Signaling in a Molecularly Distinct Subclass of Aggressive Prostate Cancer**. *J. Natl. Cancer Inst.* 2008, **100**:815-825.

9. Tomlins SA, Rhodes DR, Perner S, et al.: **Recurrent Fusion of TMPRSS2 and ETS Transcription Factor Genes in Prostate Cancer**. *Science* 2005, **310**:644-648.

10. Demichelis F, Fall K, Perner S, et al.: **TMPRSS2:ERG gene fusion associated with lethal prostate cancer in a watchful waiting cohort**. *Oncogene* 2007, **26**:4596-4599.

11. Perner S, Demichelis F, Beroukhim R, et al.: **TMPRSS2:ERG Fusion-Associated Deletions Provide Insight into the Heterogeneity of Prostate Cancer**. *Cancer Res* 2006, **66**:8337-8341.

12. Mertz KD, Setlur SR, Dhanasekaran SM, et al.: **Molecular Characterization of TMPRSS2-ERG Gene Fusion in the NCI-H660 Prostate Cancer Cell Line: A New Perspective for an Old Model**. *Neoplasia (New York, N.Y.)* 2007, **9**.

13. Livak KJ, Schmittgen TD: **Analysis of relative gene expression data using real-time quantitative PCR and the 2(-Delta Delta C(T)) Method**. *Methods* 2001, **25**:402-8.

14. Duda RO, Hart PE, Stork DG: *Pattern Classification*. Wiley; 2001:680.

15. Xu L, Shen SS, Hoshida Y, et al.: **Gene Expression Changes in an Animal Melanoma Model Correlate with Aggressiveness of Human Melanoma Metastases**. *Mol Cancer Res* 2008, **6**:760-769.

16. Dudoit S, Fridlyand J, Speed TP: **Comparison of Discrimination Methods for the Classification of Tumors Using Gene Expression Data**. *Journal of the American Statistical Association* 2002, **97**:77-87.

17. Vapnik VN: *Statistical Learning Theory*. Wiley-Interscience; 1998:736.

18. Agresti A: *An Introduction to Categorical Data Analysis*. 2nd edition. Wiley-Interscience; 2007:400.

19. Rousseeuw PJ: **Silhouettes: A graphical aid to the interpretation and validation of cluster analysis**. *Journal of Computational and Applied Mathematics* 1987, **20**:53-65.

20. Sorlie T, Perou CM, Tibshirani R, et al.: **Gene expression patterns of breast carcinomas distinguish tumor subclasses with clinical implications**. *Proceedings of the National Academy of Sciences* 2001, **98**:10869-10874.

21. Bhattacharjee A, Richards WG, Staunton J, et al.: **Classification of human lung carcinomas by mRNA expression profiling reveals distinct adenocarcinoma subclasses**. *Proc. Natl. Acad. Sci. U.S.A* 2001, **98**:13790-13795.

22. Golub TR, Slonim DK, Tamayo P, et al.: **Molecular Classification of Cancer: Class Discovery and Class Prediction by Gene Expression Monitoring**. *Science* 1999, **286**:531-537.

23. Gentleman R, Carey VJ, Huber W: *Bioinformatics and Computational Biology Solutions Using R and Bioconductor*. Birkhäuser; 2005:473.

24. Dave SS, Fu K, Wright GW, et al.: **Molecular Diagnosis of Burkitt's Lymphoma**. *N Engl J Med* 2006, **354**:2431-2442.

25. Tomlins SA, Mehra R, Rhodes DR, et al.: **Integrative molecular concept modeling of prostate cancer progression**. *Nat Genet* 2007, **39**:41-51.

26. Singh D, Febbo PG, Ross K, et al.: **Gene expression correlates of clinical prostate cancer behavior**. *Cancer Cell* 2002, **1**:203-209.

27. Subramanian A, Tamayo P, Mootha VK, et al.: **Gene set enrichment analysis: A knowledge-based approach for interpreting genome-wide expression profiles**. *Proceedings of the National Academy of Sciences of the United States of America* 2005, **102**:15545-15550.

28. Evans AJ, Henry PC, Van der Kwast TH, et al.: **Interobserver variability between expert urologic pathologists for extraprostatic extension and surgical margin status in radical prostatectomy specimens**. *Am J Surg Pathol* 2008, **32**:1503-12.

29. Burchardt M, Engers R, Müller M, et al.: **Interobserver reproducibility of Gleason grading: evaluation using prostate cancer tissue microarrays**. *J Cancer Res Clin Oncol* 2008, **134**:1071-8.

30. De la Taille A, Viellefond A, Berger N, et al.: **Evaluation of the interobserver reproducibility of Gleason grading of prostatic adenocarcinoma using tissue microarrays**. *Hum Pathol* 2003, **34**:444-9.

31. Nakagawa T, Kollmeyer TM, Morlan BW, et al.: **A Tissue Biomarker Panel Predicting Systemic Progression after PSA Recurrence Post-Definitive Prostate Cancer Therapy**. *PLoS ONE* 2008, **3**:e2318.

32. Yu YP, Landsittel D, Jing L, et al.: **Gene Expression Alterations in Prostate Cancer Predicting Tumor Aggression and Preceding Development of Malignancy**. *J Clin Oncol* 2004, **22**:2790-2799.

33. Lapointe J, Li C, Higgins JP, et al.: **Gene expression profiling identifies clinically relevant subtypes of prostate cancer**. *Proc Natl Acad Sci USA* 2004, **101**:811-816.

34. Glinsky GV, Glinskii AB, Stephenson AJ, Hoffman RM, Gerald WL: **Gene expression profiling predicts clinical outcome of prostate cancer**. *J. Clin. Invest.* 2004, **113**:913-923.

35. Glinsky GV, Berezovska O, Glinskii AB: **Microarray analysis identifies a death-from-cancer signature predicting therapy failure in patients with multiple types of cancer**. *J Clin Invest.*  2005, **115**:1503–1521.

# Supplementary Figures Legend

**Figure S1: Silhouette plots comparing Burkitt's lymphoma with different subclasses of Diffuse Large B-Cell lymphoma.**

**Figure S2: Silhouette plot of ERG rearranged cases.** The 87-gene signature determined in Setlur et al. [8] was used to computed the homogeneity score.

**Figure S3: Tomlins et al. data set.** A. Only stromal cell samples and PCA are here represented along the selected 287 genes. B. Heat map of the selected learning set samples, clustered based on the top common 47 ranked gene expressions.

**Figure S4: Over-representation of genes associated to Gleason score in an independent data set** **[26]**  **A.** Enrichment of genes over-expressed (left) and under-expressed (right) in “major Gleason 4 and greater” group. **B.** Enrichment of genes positively correlated (left) and negatively correlated (right) with Gleason score.

# Supplementary Tables

**Table S1**: Characteristics of the "Extreme design" cohort.

| Characteristic | Counts (%) | Extreme groups | | Fisher's exact test p-value | Odds Ratio (OR) [95%CI]) |
| --- | --- | --- | --- | --- | --- |
| *Gleason:(score)* |  | *Indolent* | *Lethal* |  |  |
| 4-6 | 83 (29.5) | 57 | 26 |  |  |
| 7 | 117 (41.6) | 50 | 67 |  |  |
| 8-10 | 81 (28.8) | 9 | 72 | 6*10^-14^ |  |
| *Age:(year)* |  |  |  |  |  |
| ≤70 | 83 (29.5) | 41 | 42 |  |  |
| >70 | 198 (70.5) | 75 | 123 | 0.08 | 1.6 [0.95,2.69] |
| *Tumor area in biopsy:(%)* |  |  |  |  |  |
| ≤5 | 91 (32.4) | 55 | 36 |  |  |
| 6-25 | 96 (34.2) | 43 | 53 |  |  |
| 26-50 | 51 (18.1) | 14 | 37 |  |  |
| >50 | 38 (13.5) | 3 | 35 | 1.9*10^-8^ |  |
| not assessable | 5 (2.4) |  |  |  |  |
| *ERG rearrangement status* |  |  |  |  |  |
| Negative | 226 (80.4) | 106 | 120 |  |  |
| Positive | 46 (16.4) | 5 | 41 | 2.3*10^-6^ | 7.2 [2.8,19.0] |
| not assessable | 9 (3.2) |  |  |  |  |
| *Extreme group:* |  |  |  |  |  |
| Lethal | 165 (58.7) |  |  |  |  |
| Indolent | 116 (41.3) |  |  |  |  |

**Table S2**: Data sets employed for the homogeneity analysis.

| **Authors** | **Title** | **Journal** | **Tumor Type** | **Sample description** |
| --- | --- | --- | --- | --- |
| Sørlie T et al. | **Gene expression patterns of breast carcinomas distinguish tumor subclasses with clinical implications** | PNAS 2001 98(19):10869-10874 | Breast cancer | 85 samples (ER-18; ER+56) |
| Golub TR et al. | **Molecular Classification of Cancer: Class Discovery and Class Prediction by Gene Expression Monitoring** | Science 1999 286(5439):531-537 | Leukemia | 38 samples (Acute Myeloid Leukemia – AML 11; Acute Lymphoblastic Leukemia – ALL: 27) |
| Bhattacharjee A et al. | **Classification of human lung carcinomas by mRNA expression profiling reveals distinct adenocarcinoma subclasses** | PNAS 2001 98(24):13790-13795 | Lung cancer | 144 samples (127 adenocarcinomas – 17 normals): Dataset B |
| Dave et al. | **Molecular Diagnosis of Burkitt's Lymphoma** | N Engl J Med 2006 354(23):2431-2442 | Lymphoma | 303 samples (41 Burkitt's lymphoma – BL; 257 Diffuse large B-cell lymphoma – DLBCL) |

**Table S3: Cross-Validation results of the classification models on the Learning set**. The table reports the AUC for the best models of each algorithm type and its optimal parameters.

| **Algorithms** | **Parameters of the best model** | **AUC** | **95%CI** |
| --- | --- | --- | --- |
| SVM (Polynomial kernel) | P = 0.001 Degree = 1, Cost = 0.1 | 0.786 | 0.758-0.814 |
| DLDA | 133 predictors, P = 0.01 | 0.770 | 0.740-0.800 |
| LR | 18 predictors, P = 0.01 | 0.738 | 0.694-0.781 |
| NN | P = 0.01, Decay=0.1, Size=3 | 0.730 | 0.634-0.826 |
| NTP | P=0.005 | 0.716 | 0.710-0.723 |
| kNN | P=0.005, k=5 | 0.707 | 0.700-0.714 |

**Table S4: Gene list for the logistic regression models reported in Figure 2**. The table shows the gene symbol, its description, and location. Then, it reports the number and which models the genes was selected. The models are separated with the pipe ('|') symbol, and report the number of genes and the clinical parameters. For example 9-age-ERG represents the model with 9 genes, age at diagnosis and ERG rearrangement positive as predictor. Models with no clinical parameters are indicated by 'noClinical'.

| **Symbol** | **Description** | **location** | **#models** | **models** |
| --- | --- | --- | --- | --- |
| MDK | midkine (neurite growth-promoting factor 2) | 11p11.2 | 9 | 11-age|12-gleason_age|12-gleason_age_ERG|16-gleason|18-ERG|18-noClinical|21-gleason_ERG|3-gleason_age_tum.perc|9-age_ERG |
| SHMT2 | serine hydroxymethyltransferase 2 (mitochondrial) | 12q12-q14 | 8 | 11-age|12-gleason_age|12-gleason_age_ERG|16-gleason|18-ERG|18-noClinical|21-gleason_ERG|9-age_ERG |
| PIP5K1A | phosphatidylinositol-4-phosphate 5-kinase, type I, alpha | 1q22-q24 | 8 | 11-age|12-gleason_age|12-gleason_age_ERG|16-gleason|18-ERG|18-noClinical|21-gleason_ERG|9-age_ERG |
| PLA2G7 | phospholipase A2, group VII (platelet-activating factor acetylhydrolase, plasma) | 6p21.2-p12 | 8 | 11-age|12-gleason_age|12-gleason_age_ERG|16-gleason|18-ERG|18-noClinical|21-gleason_ERG|9-age_ERG |
| HSF1 | heat shock transcription factor 1 | 8q24.3 | 7 | 12-gleason_age|12-gleason_age_ERG|16-gleason|18-ERG|18-noClinical|21-gleason_ERG|9-age_ERG |
| SLC1A1 | solute carrier family 1 (neuronal/epithelial high affinity glutamate transporter, system Xag), member 1 | 9p24 | 6 | 12-gleason_age|12-gleason_age_ERG|16-gleason|18-ERG|18-noClinical|21-gleason_ERG |
| BMPER | BMP binding endothelial regulator | 7p14.3 | 5 | 12-gleason_age|12-gleason_age_ERG|16-gleason|21-gleason_ERG|3-gleason_age_tum.perc |
| HSD17B6 | hydroxysteroid (17-beta) dehydrogenase 6 homolog (mouse) | 12q13 | 5 | 12-gleason_age|12-gleason_age_ERG|16-gleason|21-gleason_ERG|3-gleason_age_tum.perc |
| MYBPC1 | myosin binding protein C, slow type | 12q23.2 | 4 | 11-age|18-ERG|18-noClinical|9-age_ERG |
| KCNJ5 | potassium inwardly-rectifying channel, subfamily J, member 5 | 11q24 | 4 | 12-gleason_age|12-gleason_age_ERG|16-gleason|21-gleason_ERG |
| CAND1 | cullin-associated and neddylation-dissociated 1 | 12q14 | 4 | 11-age|18-ERG|18-noClinical|9-age_ERG |
| FOLH1 | folate hydrolase (prostate-specific membrane antigen) 1 | 11p11.2 | 4 | 16-gleason|18-ERG|21-gleason_ERG|9-age_ERG |
| GLS | glutaminase | 2q32-q34 | 4 | 12-gleason_age|12-gleason_age_ERG|16-gleason|21-gleason_ERG |
| AP1B1 | adaptor-related protein complex 1, beta 1 subunit | 22q12 ; 22q12.2 | 3 | 12-gleason_age_ERG|18-ERG|21-gleason_ERG |
| FOXD1 | forkhead box D1 | 5q12-q13 | 3 | 12-gleason_age|16-gleason|21-gleason_ERG |
| GRIA3 | glutamate receptor, ionotrophic, AMPA 3 | Xq25-q26 | 3 | 18-ERG|18-noClinical|21-gleason_ERG |
| UNC119 | unc-119 homolog (C. elegans) | 17q11.2 | 2 | 18-ERG|21-gleason_ERG |
| NME3 | non-metastatic cells 3, protein expressed in | 16q13 | 2 | 18-ERG|21-gleason_ERG |
| PDCD6 | programmed cell death 6 | 5pter-p15.2 | 2 | 16-gleason|21-gleason_ERG |
| MCM4 | minichromosome maintenance complex component 4 | 8q11.2 | 2 | 18-ERG|18-noClinical |
| ENO1 | enolase 1, (alpha) | 1p36.3-p36.2 | 2 | 12-gleason_age_ERG|21-gleason_ERG |
| FMO5 | flavin containing monooxygenase 5 | 1q21.1 | 2 | 11-age|18-noClinical |
| BCAS1 | breast carcinoma amplified sequence 1 | 20q13.2-q13.3 | 2 | 11-age|18-noClinical |
| EYA1 | eyes absent homolog 1 (Drosophila) | 8q13.3 | 2 | 11-age|18-noClinical |
| RPE65 | retinal pigment epithelium-specific protein 65kDa | 1p31 | 2 | 11-age|18-noClinical |
| SLCO2A1 | solute carrier organic anion transporter family, member 2A1 | 3q21 | 2 | 12-gleason_age|16-gleason |
| GP1BB | glycoprotein Ib (platelet), beta polypeptide | 22q11.21-q11.23 ; 22q11.21 | 2 | 11-age|18-noClinical |
| EPHX2 | epoxide hydrolase 2, cytoplasmic | 8p21-p12 | 1 | 18-noClinical |
| ALOX15B | arachidonate 15-lipoxygenase, type B | 17p13.1 | 1 | 18-noClinical |
| GSTT2 | glutathione S-transferase theta 2 | 22q11.2 ; 22q11.23 | 1 | 9-age_ERG |
| GFPT2 | glutamine-fructose-6-phosphate transaminase 2 | 5q34-q35 | 1 | 21-gleason_ERG |
| DAZAP2 | DAZ associated protein 2 | 12q12 | 1 | 21-gleason_ERG |
| HOXB13 | homeobox B13 | 17q21.2 | 1 | 21-gleason_ERG |
| SERPINA3 | serpin peptidase inhibitor, clade A (alpha-1 antiproteinase, antitrypsin), member 3 | 14q32.1 | 1 | 18-noClinical |
| ISL1 | ISL1 transcription factor, LIM/homeodomain, (islet-1) | 5q11.2 | 1 | 16-gleason |
| CRIP2 | cysteine-rich protein 2 | 14q32.3 | 1 | 18-ERG |
| SNCA | synuclein, alpha (non A4 component of amyloid precursor) | 4q21 | 1 | 18-ERG |
| PTK6 | PTK6 protein tyrosine kinase 6 | 20q13.3 | 1 | 16-gleason |
| SERTAD2 | SERTA domain containing 2 | 2p14 | 1 | 18-ERG |
| PTK7 | PTK7 protein tyrosine kinase 7 | 6p21.1-p12.2 | 1 | 18-ERG |

**Table S5: List of 118 differentially expressed genes between Lethal and Indolents.** A q-value (false discovery rate) of 0.05 on a two-tailed t-test was used to select these genes. These genes were used for the homogeneity analysis of the prostate cancer set shown in Figure 4.

| **Genes** | | |
| --- | --- | --- |
| MYBPC1 | PTK7 | CDC42BPA |
| PLA2G7 | CACNA1D | CRIP2 |
| BCAS1 | RGS4 | NPAL3 |
| SERPINA3 | PDCD6 | FOXD1 |
| SEMA3F | PSMA7 | ACTB |
| EHHADH | SLCO2A1 | GFPT2 |
| SHMT2 | ECE1 | NOTCH3 |
| EYA1 | MT2A | GARS |
| GP1BB | DLGAP1 | TRIP13 |
| RPE65 | ABAT | MDK |
| SLC1A1 | PDE9A | JAG1 |
| FMO5 | EGF | FAM129A |
| GMDS | SCUBE2 | ATP8A2 |
| ITPR2 | BIRC5 | EPHX2 |
| HSD17B6 | HDAC1 | UPK2 |
| NDRG1 | BMPER | MT1X |
| PMS2L3 | XRCC2 | PRKCB1 |
| ALOX15B | METTL7A | REPS2 |
| MCM4 | HLXB9 | ADH5 |
| SH3BGRL | COL4A1 | RGS5 |
| PRKAR1B | COL9A2 | CYC1 |
| GRIA3 | TGFB2 | CPT1A |
| CAND1 | INHBA | BAIAP2 |
| MT1G | UBAP2L | PLCG1 |
| SLC15A2 | KHDRBS3 | SECISBP2 |
| STIP1 | PTPRM | CYP2J2 |
| BGN | KCNN2 | PEX10 |
| SATB1 | LAMC1 | TFDP1 |
| SMPDL3A | C2 | PRKCZ |
| FOLH1 | TOP2A | EIF4G3 |
| GPR116 | FAM110B | LMNB1 |
| SLC39A8 | GPR137B | ATP5B |
| RAB27A | OCLN | PRDX6 |
| TLE1 | BANK1 | ERG |
| KCNMA1 | AZGP1 | VEGFA |
| AP1B1 | CHRNA2 | PFKFB1 |
| MPPED2 | TFF3 | MTA1 |
| UNG | CDK6 | VHL |
| MT1F | YWHAZ |  |
| INPP4B | PRR4 |  |

**Table S6: Gleason score for Swedish and Singh datasets**.

|  | ***Swedish*** | | ***Singh*** | | |
| --- | --- | --- | --- | --- | --- |
| Gleason | N | % | N | % | |
| 5 | 0 | 0% | 4 | 8% | |
| 6 | 110 | 30% | 15 | 29% | |
| 7 | 153 | 42% | 29 | 56% | |
| 8 | 41 | 11% | 2 | 4% | |
| 9 | 54 | 15% | 2 | 4% | |
| 10 | 5 | 1% | 0 | 0% | |
| Total | 363 |  | 52 |  |  |

**Table S7: Cross-validated results for DLDA after modifying the definition of "extreme" cases.** Results of the best model only are reported. The first column shows the new definition of lethal and indolents with the number of samples in that category in parenthesis.

| Extreme case redefinition | AUC | Num. genes |
| --- | --- | --- |
| All lethal (n=110) vs All Indolents (n=76) | 0.77 | 133 |
| Lethal < 5yr (n=55) vs All Indolents (n=76) | 0.83 | 20 |
| Lethal < 5yr (n=55) vs Indolent >= 13yr (n=36) | 0.77 | 16 |
| Lethal <= 8yr (n=86) vs All Indolents (n=76) | 0.75 | 33 |
| Lethal <= 8yr (n=86) vs Indolent >= 11yr (n=50) | 0.82 | 66 |

1. This definition holds for sample partitions made by two groups only. When samples are classified in more than two groups, *b_i_* is computed in the same way as in the two-group case for each group and then the minimum value is considered. [↑](#footnote-ref-2)
