# Supplementary material for: Molecular sampling of prostate cancer: a dilemma for predicting disease progression
Source: BMC Med Genomics. 2010 Mar 16;3:8. doi: 10.1186/1755-8794-3-8 (PMC2855514; doi:10.1186/1755-8794-3-8)
Supplement: Additional file 2 — Supplementary figures. This file contains supplementary figures. [file 1755-8794-3-8-S2.PDF]

Silhouette Plots

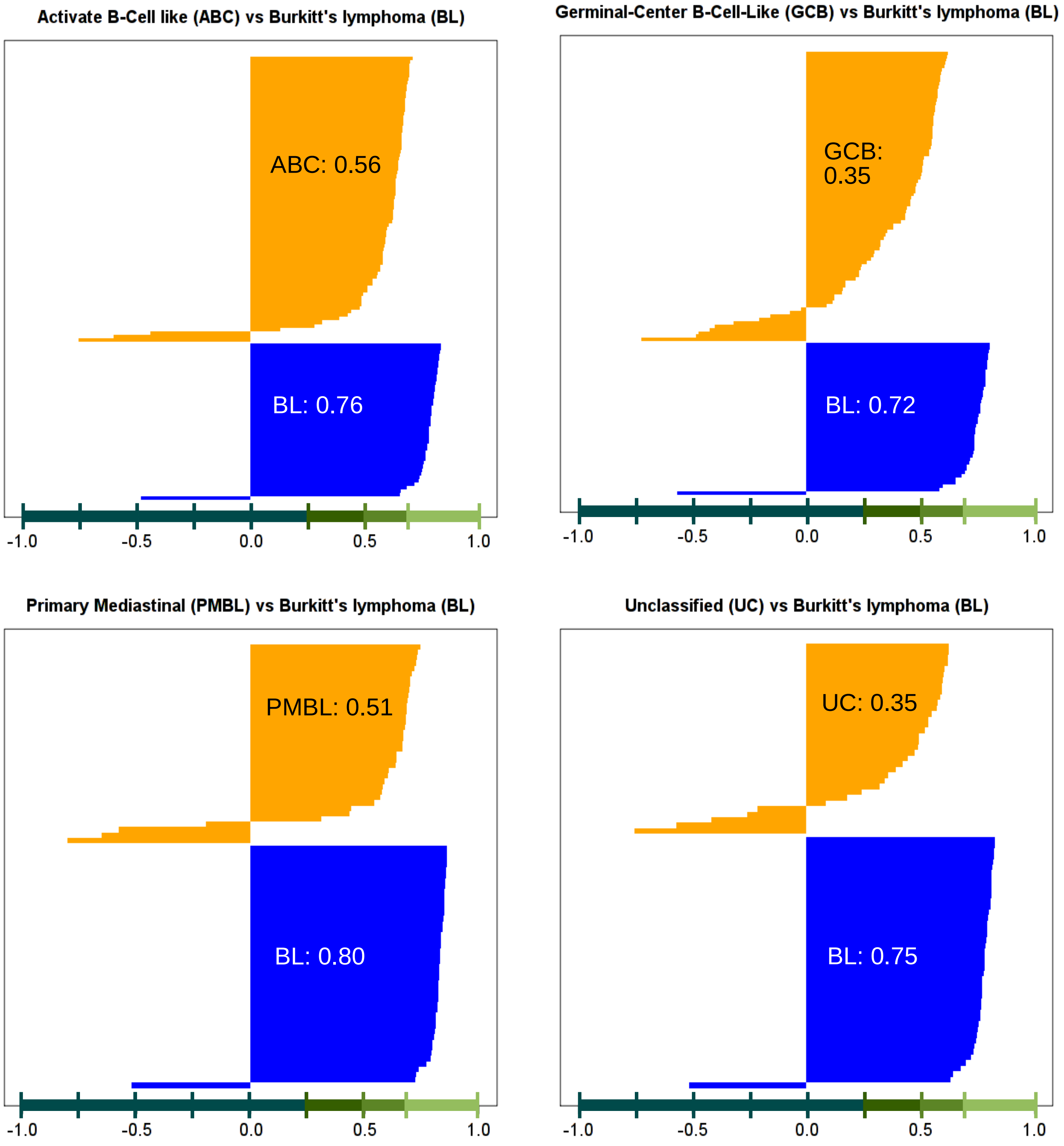

Suppl Figure S1

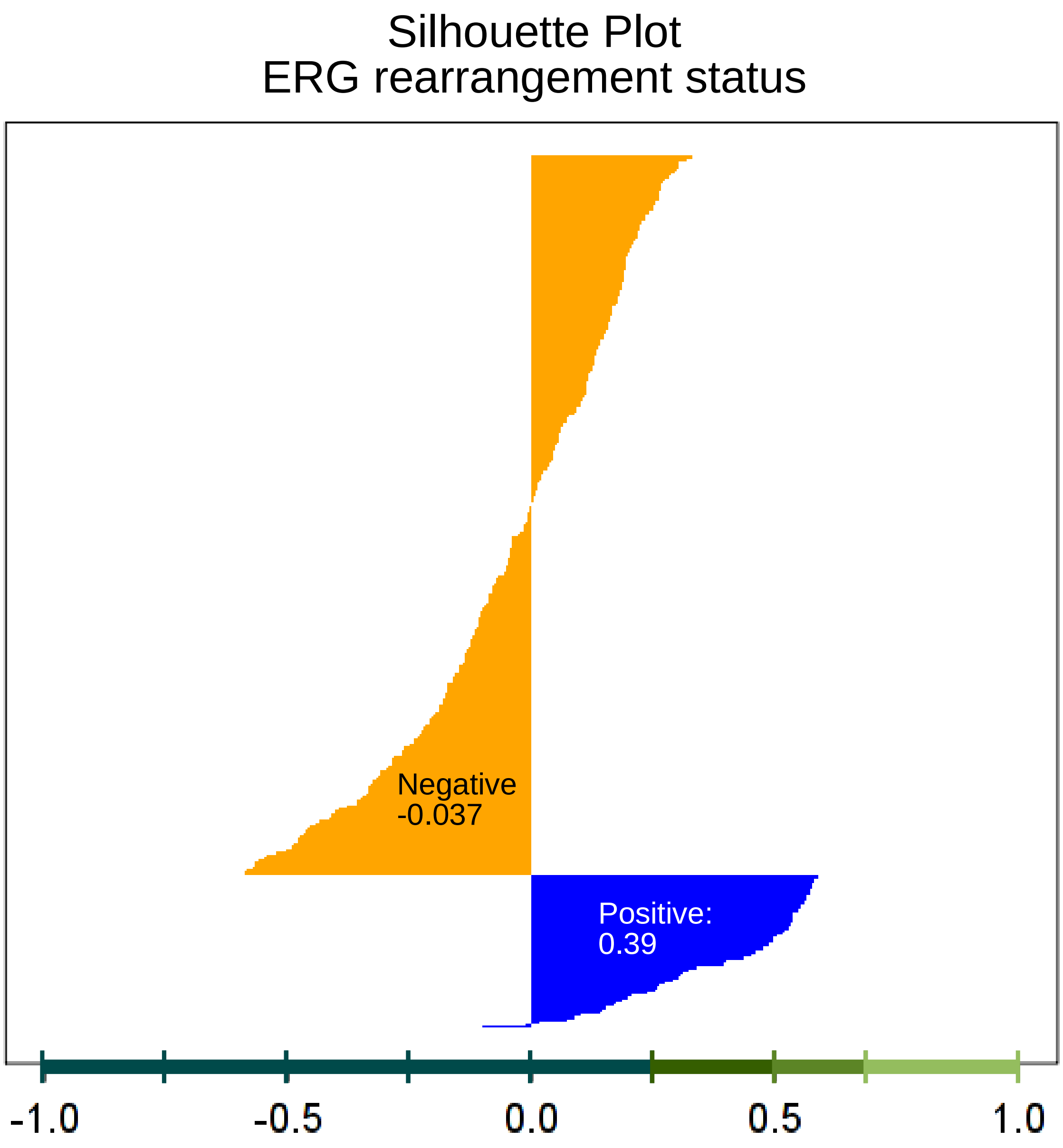

Suppl Figure S2

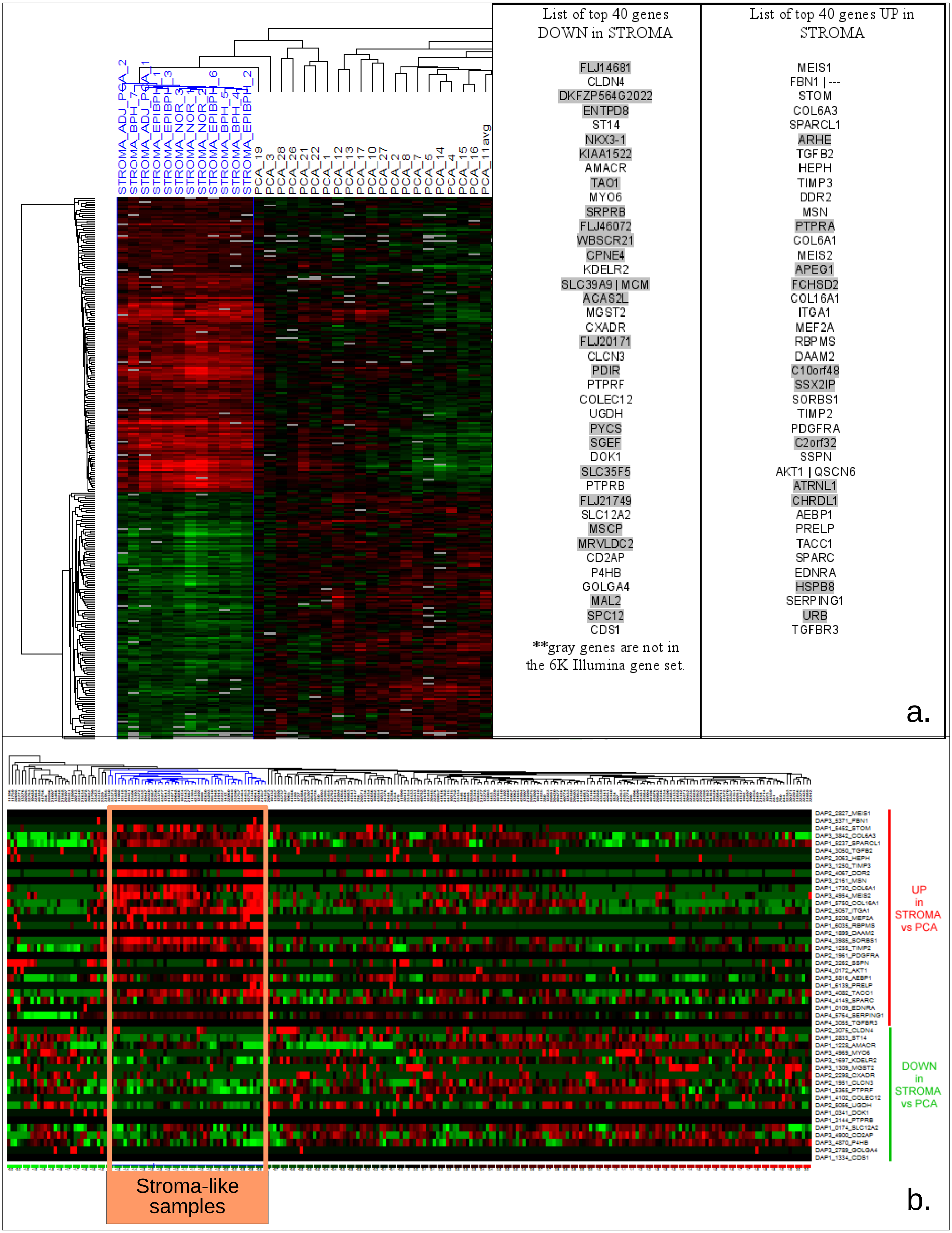

Suppl Figure S3

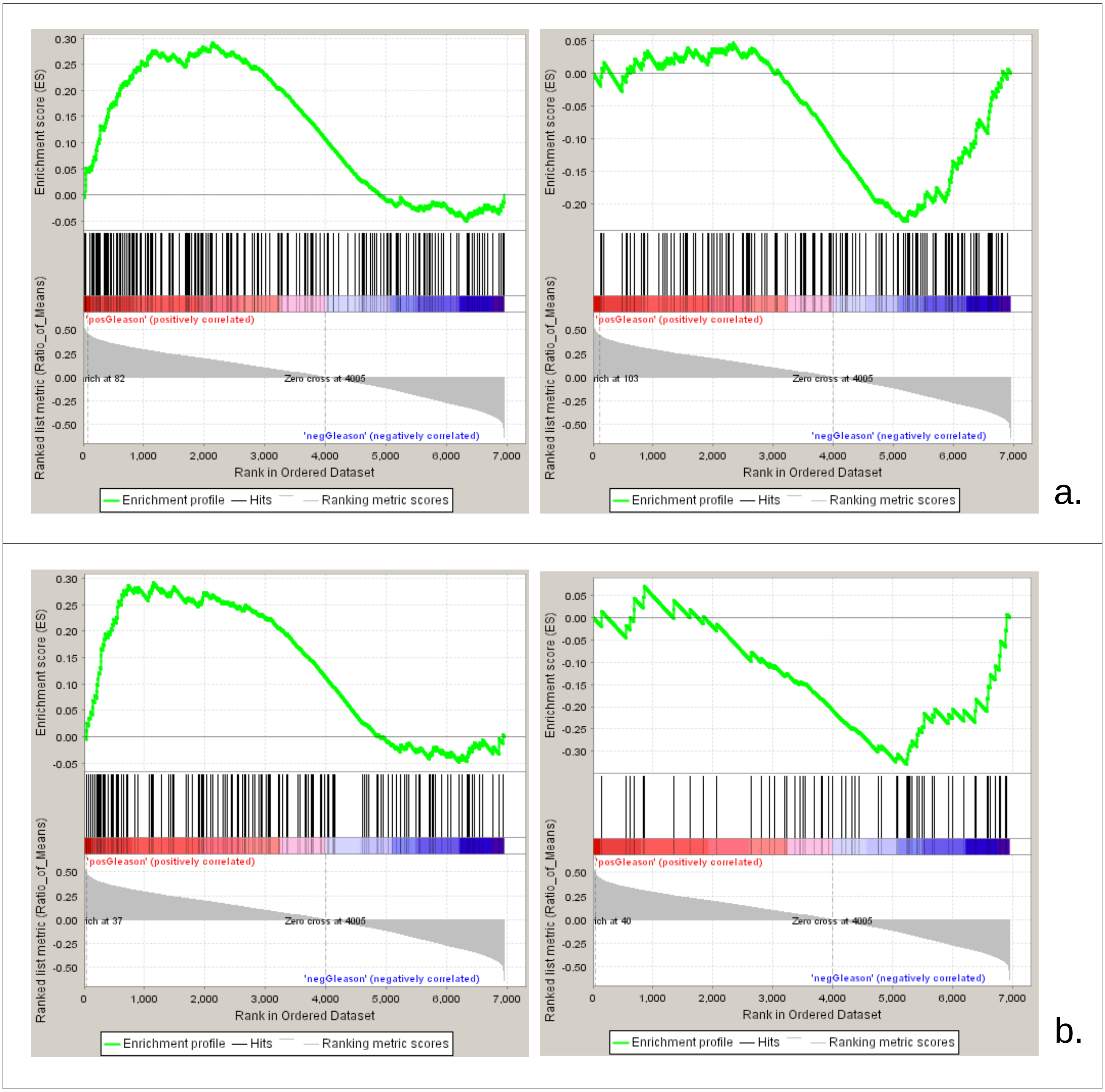

Suppl Figure S4
